# Plasma derived extracellular vesicle biomarkers of microglia activation in an experimental stroke model

**DOI:** 10.1186/s12974-023-02708-x

**Published:** 2023-01-31

**Authors:** A. D. Roseborough, S. J. Myers, R. Khazaee, Y. Zhu, L. Zhao, E. Iorio, F. M. Elahi, S. H. Pasternak, S. N. Whitehead

**Affiliations:** 1 Vulnerable Brain Laboratory, Department of Anatomy and Cell Biology, The Schulich School of Medicine and Dentistry, The University of Western Ontario, 458 Medical Sciences Building, ON N6A 3K London, Canada; 2 Biotron Integrated Microscopy Facility, The University of Western Ontario, London, ON Canada; 3 Deparment of Biology, The University of Western Ontario, London, ON Canada; 4 Weill Institute for Neurosciences, University of California San Francisco, San Francisco, CA USA; 5 Icahn School of Medicine at Mount Sinai, New York, USA; 6 Department of Clinical Neurological Sciences, The Schulich School of Medicine and Dentistry, The University of Western Ontario, ON London, Canada; 7 Robarts Research Institute, The Schulich School of Medicine and Dentistry, The University of Western Ontario, ON London, Canada

**Keywords:** Microglia, Stroke, Extracellular vesicle

## Abstract

Chronic microglia activation post-stroke is associated with worse neurological and cognitive outcomes. However, measurement of microglia activation in vivo is currently limited. Plasma derived extracellular vesicles (EVs) are cell-specific indicators that may allow for non-invasive measurement of microglia phenotype. The aim of this study was to identify activation-state specific microglia EVs (MEVs) in vitro followed by validation in an experimental stroke model. Following pro-inflammatory activation, MEVs contain the microglia protein TMEM119 alongside increased expression of the Toll-like receptor 4 co-receptor CD14. Immunoprecipitation followed by fluorescent nanoparticle tracking analysis (ONI Nanoimager) was used to confirm the isolation of TMEM119^+^/CD14^+^ EVs from rat plasma. Electron microscopy confirmed that TMEM119 and CD14 localize to the MEV membrane. To model ischemia, plasma was collected from 3-month wildtype Fischer344 rats prior to, 7 and 28 days after endothelin-1 or saline injection into the dorsal right striatum. Fluorescently labelled MEVs were directly measured in the plasma using nanoflow cytometry (Apogee A60 Microplus). We report a significant increase in circulating TMEM119^+^/CD14^+^ EVs 28-days post-stroke in comparison to baseline levels and saline-injected rats, which correlated weakly with stroke volume. TMEM119^+^/MHC-II^+^ EVs were also increased post-stroke in comparison to baseline and saline-injected animals. This study is the first to describe an EV biomarker of activated microglia detected directly in plasma following stroke and represents a future tool for the measurement of microglia activity in vivo*.*

**Supplementary Information:**

The online version contains supplementary material available at 10.1186/s12974-023-02708-x.

## Introduction

Microglia represent the resident macrophagic cells of the brain and play a crucial role in the response to acute neurological injuries including traumatic brain injury, hemorrhage, and stroke [1, 2]. As a highly dynamic cell type, microglia exhibit context-dependent phenotypes characterized by transcriptional profiles, surface marker expression, and the release of signalling molecules [3–6]. In response to ischemic stroke, microglia rapidly proliferate and represent the dominant cell type within the stroke-induced lesion despite infiltration from peripheral macrophages [7, 8]. In the acute stage, microglia initially adapt an amoeboid phenotype characterized by the release of neurotrophic factors and phagocytosis of extracellular debris [9–11]. In addition to their phagocytic activity, post-stroke microglia can exhibit a neurotoxic activation state involving the release of pro-inflammatory cytokines, chemokines and reactive oxygen species [12, 13]. If unresolved, chronic activation of pro-inflammatory microglia in the weeks and months following stroke impairs neurogenesis, increases amyloid and iron deposition and reduces long-term potentiation of synapses. These consequences have detrimental effects on the mitigation of tissue damage and promotion of functional recovery [14–18].

Previous work in both preclinical and clinical studies have demonstrated that a chronic post-stroke increase of pro-inflammatory microglia promotes worse neurological outcomes and neurodegeneration secondary to the acute insult [14, 19–22]. This is further supported by studies demonstrating the beneficial effects of anti-inflammatory treatments designed to reduce chronic microglia activation on lesions size, neurological outcomes and circulating inflammatory molecules [23–25]. Despite the recognized consequences of chronic pro-inflammatory microglial activity, tracking microglia in vivo remains limited by the inability to readily biopsy human brain tissue, and the lack of specificity of current positron emission tomography (PET) tracers of microglia [26, 27]. Although circulating pro-inflammatory molecules measured using ELISA or other molecular techniques are increased post-stroke, these readouts are not specific to the central nervous system (CNS) and provide no direct information on CNS microglia activity. Therefore, non-invasive, and specific approaches are required for the accurate tracking of microglia activity in the chronic stages of neurological injury such as stroke.

Brain-derived extracellular vesicles (EVs) have emerged as biomarkers for neurological diseases given their ability to cross the blood–brain barrier and be isolated from the peripheral circulation; along with their expression of cell-specific proteins [28]. Unique profiles of circulating EV markers have been recently identified in the plasma of individuals with white matter aging [29, 30], and neurodegenerative diseases including Alzheimer’s disease, mild cognitive impairment, and Parkinson’s disease [31–35]. However, the use of EVs as indicators of pathophysiological processes post-stroke remains understudied. Furthermore, prior studies of brain derived EVs have focused primarily on neuronal, astrocytic, or endothelial cell derived EVs whilst circulating microglia derived EVs (MEVs) have not been well described.

EVs released from microglia bear the characteristic microglia proteins TMEM119 and Iba1, can be detected in the cerebrospinal fluid, and represent large contributors to the brain derived EV pool in healthy individuals [36]. Furthermore, in vitro studies have demonstrated that MEVs are altered upon pro-inflammatory stimuli, displaying surface proteins and cargo reflective of microglia activation state [37]. This suggests that given the dynamic nature of microglia phenotype post-stroke, MEVs have promise as indicators of cellular activity. To date, detection of MEVs in plasma and the identification of phenotype specific MEVs in animal or human samples has not been reported. To address this outstanding question, we investigated MEV signatures of microglia activation in vitro and validated them in baseline, subacute (7-day) and chronic (28-day) plasma samples from an experimental rat stroke model. Pro-inflammatory MEVs were detected and measured in the plasma using nanoflow cytometry, representing the first study to our knowledge to directly measure circulating MEVs without prior EV enrichment steps. We report detection of phenotype- specific surface proteins, CD14 and antigen presenting MHC Class II, on TMEM119^+^ MEVs that reflect upregulated pro-inflammatory microglia activation. Plasma detection of phenotype specific MEVs represents a non-invasive, simple method of assessing microglia activity with translational relevance for post-stroke monitoring and applicability to other acute neurological injuries and disease.

## Methods

### Animals and surgical procedures

Sixteen 3-month-old male Fischer344 rats were randomly assigned to either surgery (*n* = 8) or sham (*n* = 8) groups. Animals were anesthetized using 3% isoflurane and maintained at 1.5% isoflurane with body temperature maintained at 37 °C. Either saline (3 µL) or saline containing Endothelin-1 (600 pmol in 3 µL) was administered using stereotaxic injection into the right dorsal striatum.

### Blood collections

Blood (500 µL) was collected from the tail-vein prior to surgery and again 7 days following surgery. Terminal 28-day blood was collected from the left ventricle at the time of euthanasia Blood was collected using an 18-gauge needle and lithium heparin coated Microvette 500 tubes (Sarstedt) and plasma was isolated within 30-min using two rounds of centrifugation at 2500×*g* for 15 min at 4 °C, aliquoted and stored at − 80 °C prior to nanoflow analysis. After the first round of centrifugation, supernatant was collected above the buffy coat and transfer to a clean 1.5 ml tube for the second spin.

### Nanoflow cytometry

Ten µL plasma was incubated with combinations of the following antibodies at room temperature for 30 min: anti-TMEM119 Coralite-647 (100 ng/sample, Proteintech 66948), anti-CD14 Alexa Fluor-488 (100 ng/sample, Bioss 1192R) and anti-MHC-II (OX-6) (50 ng/sample), without any wash steps after incubation. All samples from the same animal were run on the same day to minimize variability, with triplicate antibody incubations for each sample (3 × 10 µL). All incubations had a final concentration of 0.0125% Triton-X-100 to permeabilize EVs and minimize lysis [38]. After incubation, samples were diluted 50-fold (for a final dilution of 100-fold) and triplicates of each incubation were run on the Apogee A50 Microplus Nanoflow Cytometer (Apogee Flow Systems Inc). Instrument settings to achieve data linearity using the Apogee nanoflow cytometer have been previously published and followed in this study [39]. Prior to running plasma samples, it was ensured that background levels of buffer (PBS) did not exceed 100 eV/s and standardized beads were run to confirm they were measured at their stock concentration of 5000 eV/s. Instrument settings: Sheath pressure: 150 mbar, flow rate: 150 µL/min for 130 µL, lasers: 100 mW 405 nm (violet), 70mW 638 nm (red), 70 mW 488 (green). Light scatter of events was produced using the 405 nm laser, with thresholds to eliminate background noise of 34 a.u. for small angle light scatter (SALS) and 21 a.u. for long angle light scatter (LALS). Photomultiplier tube (PMT) voltages: LALS (265 V) SALS (340 V) L488-Grn (525 V) L638-Red (650). Additional file 1: Fig. S1 includes polystyrene and silicon sizing beads for comparison of EV sizes. Nanoflow cytometry of dilution reagent (PBS), single antibodies and unlabeled plasma are reported in Additional file 1: Fig. S1. Outputs of EV numbers are reported as events/µl which represents the concentration of labelled particles detected after gating by fluorescent channel of antibodies used.

### Cell culture

Cell growing conditions: BV-2 cells were donated from Dr. Tuan Trang at the University of Calgary. BV-2 cells were cultured at 37 °C in a humidified atmosphere with 5% CO_2_. Cells were maintained in Dulbecco’s Modified Eagle Medium (Gibco) supplemented with 10% fetal bovine serum, Penicillin (100 units/mL) and Streptomycin (100 μg/mL) (Gibco). Lipopolysaccharide (LPS) exposure: Cells were seeded in 24 well plates (Thermo Fisher Scientific) at 1.7 × 10^5^ cells/well for RNA collection, 5 × 10^4^ cells/well for immunofluorescent staining, or T75 flasks (Thermo Fisher Scientific) for EV collection. 8 h prior to treatment cells were washed with PBS and switched to serum free media. Cells were treated with LPS (Sigma) at 100 ng/mL and 500 ng/mL for 4–24 h. Primary adult microglia: Cells were isolated using a modified version of a protocol adapted from Agalave et al., 2020 that has been previously described [40]. Modifications included: a 75% Percoll gradient was substituted instead of 70%, rat brains were divided into two gradients per brain, tissue was homogenized mechanically and passed through a 100 µm cell strainer prior to the 70 μm cell strainer, cells were seeded at a density of 150,000 cells per well of 24-well plate.

### Molecular analyses

RNA isolation: After 4-, 8- or 12-h RNA was extracted using a TRIzol (Life Technologies). Samples were brought up to 600 μL Trizol followed by the addition of chloroform (20% of volume). Following vigorous mixing samples were centrifuged at 12,000×*g* for 15 min at 4 °C. The aqueous phase was retained and mixed by inversion with an equal volume of isopropyl alcohol followed by a 30-min incubation at − 20 °C. Samples were then centrifuged at 12,000×*g* for 10 min at 4 °C after which the supernatant was removed. The resultant pellet was washed with 75% ethanol and resuspended in 20 µL RNase-free water after air drying. Following extraction, RNA concentrations were determined using a Nanodrop One spectrophotometer (Thermo Fisher Scientific). Quantitative real time PCR (qPCR): cDNA was synthesized using 2 μg of RNA from each sample. Target gene amplification was performed with specific forward and reverse primers designed using the NCBI primer design tool. Primer sequences and GenBank accession numbers are provided in Additional file 1: Table S1. 2 µL of cDNA was combined with forward and reverse primers (at 125 μM each) and SsoAdvanced Universal SYBR Green Mix (Bio-Rad). RPL13α was used as an endogenous control with all mRNA expression levels normalized to this value. RPL13α was chosen as it demonstrated stable expression across treatment groups and has previously been used in studies of microglia pro-inflammatory signaling [41, 42]. Comparison of transcription levels was performed using the ΔΔC^T^ method [43].

### EV isolation and transmission electron microscopy (TEM)

Following 24 h of LPS exposure, EVs were isolated from cell culture media for western blot and transmission electron microscopy (TEM). Plasma-derived EVs were isolated from 250 µL post-stroke rat plasma. Media/plasma was centrifuged at 15,000×*g* for 15 min at 4 °C to remove cellular debris, filtered through 0.2 μm SFCA syringe filters (Thermo Fisher Scientific) and concentrated using 10 kDa centrifugation filters (Amicon Ultra-15). Concentrated media was ultracentrifuged at 100,000×*g* for 1 h and 15 min at 4 °C. For western blot: The EV pellet was washed once with PBS prior to resuspension, and the concentration of EVs (ev/uL) was determined using nanoflow cytometry. 1 × 10^9^ EVs per sample were used for subsequent western blotting. For TEM: the EV pellet was resuspended in 2% paraformaldehyde and 0.1% glutaraldehyde in 0.1 M sodium cacodylate buffer (pH 7.0) and fixed for 1 h at 4 °C. EVs were pelleted again via ultracentrifugation at 100,000×*g* at 4 °C for 1 h and 15 min. EV pellet was then washed three times for 10 min each with 0.1 M sodium cacodylate buffer. EV pellet was stained for visualization with 0.2% osmium tetroxide for 1 h at 4 °C, washed three times for 10 min each with double-distilled water (ddH_2_0), and dehydrated in an ascending series of acetone solutions. EV pellet was embedded in LR white resin at 50 °C for 24 h. Ultra-thin (90 nm) sections were cut (Ultramicrotome Reichert-Jung Ultracut E; Leica Microsystems, Wetzlar, Denmark) and collected on nickel grids. Free aldehyde groups were quenched using 0.02 M glycine for 10 min, followed by three times 10-min rinses with ddH_2_O. Sections were blocked with 1% BSA in 1 × phosphate buffer saline (PBS) prior to incubation with primary antibody for 1 h at room temperature. Sections were washed with 0.1% BSA in PBS prior to incubation with gold-conjugated secondary antibody for 1 h. Sections were rinsed five times with 0.1% BSA in 1 × PBS. Finally, Sections were rinsed two times with ddH_2_O prior to visualization. Imaging of isolated EVs was carried out using a Transmission Electron Microscope CM10 (Philips Electron Optics, Eindhoven, The Netherlands). Wide-field images of unlabeled and CD14 or TMEM119 labeled EVs are provided in Additional file 1: Figure S3.

### Immunoprecipitation

50 μg of plasma derived EVs was incubated with TMEM119 (1:50) in 200 μL PBS overnight at 4 °C. Following incubation, 50% protein A/G bead slurry (Pierce) was added and incubated for one hour with rotation at 4 °C. The samples were then centrifuged for 30 s 4 °C, and the pellet was washed 5 times with 500 μL PBS. After the final wash the pellet was resuspended in loading buffer, vortexed, centrifuged for 30 s at 1400×*g*, denatured at 95 °C for 5 min and centrifuged for one minute at 1400×*g* prior to western blotting.

### Dual immunoprecipitation

MEVs were enriched by sequential immunoprecipitation with a protocol adapted from Elahi et al. 2021. Briefly, the total double selected microglial EVs were enriched by sequential immunoprecipitation using two biotinylated monoclonal antibodies mouse anti-human Transmembrane Protein 119 (TMEM119) (Novus Biologicals, LLC., Centennial, CO, USA) and then mouse anti-human CD14 biotinylated antibody (Sigma-Aldrich Co LLC).

### Nanoparticle tracking analysis/single-molecule tracking

EVs were labeled with ExoGlow membrane EV labeling kit (Cat# EXOGM600A-1, System Biosciences, Palo Alto, CA) according to the manufacturer’s instructions. EV concentration and size distribution were analyzed via single-molecule tracking using the ONI Nanoimager (Oxford Nanoimaging; ONI) with a 488-nm wavelength laser at an exposure of 10 ms for 5000 captured frames.

### Western blot

Concentration of EV isolated was determined using nanoflow cytometry and 1 × 10^9^ EVs were loaded per lane. EVs were diluted in loading buffer (1 × LDS, 5 mM DTT in 0.5% SDS). Proteins were denatured for 10 min at 70 °C and separated using gel electrophoresis of Bis–Tris 10% acrylamide gels in MOPS SDS running buffer (Thermo Fisher Scientific) at 70 mA per gel, followed by transfer to a PVDF membrane (Roche Diagnostics) at 100 V for 100 min on ice. After transfer the membrane was washed briefly in TBST (0.1% Tween) and blocked overnight at 4 °C with 5% milk in TBST. After blocking the membranes were washed briefly in Tris Buffered Saline-Tween 20 (TBST) (Tris–HCl 50 mM pH 8.0, NaCl 0.15 M, Tween 20 0.1% v/v) and incubated with the following primary antibodies in 5% BSA in TBST overnight at 4 °C: TMEM119 (1:1000, ProteinTech 66948), CD14 (1:1000, Bioss 1192-R), Iba1 (1:1000, Wako 019-19741), CD9 (1:1000, Abcam ab223052) CD13 (1:1000,Abcam ab108310), TSG101 (1:1000, Abcam ab03871). TSG101 was used as a loading control and for normalization of protein for quantification. Membranes were washed 3 × for 10 min with TBST and incubated with donkey anti-rabbit or donkey anti-mouse HRP-conjugated secondary antibodies (1:10,000, Jackson Laboratories) for one hour at room temperature. Finally, membranes were washed 3 × for 10 min with TBST prior to detection with chemiluminescent HRP substrate (Immobilon) and imaging using a ChemiDoc MP system (Bio-Rad).

### Euthanasia and brain collection

Animals were euthanized 28 days post-surgery using an intraperitoneal injection of pentobarbital and transcardial perfusion of 180 mL 0.01 M PBS followed by 300 mL 4% paraformaldehyde (PFA) (pH 7.4). Brains were removed and stored in 4% PFA for 24 h, transferred to 30% sucrose for 36 h at 4 °C prior to being cut into either 10 or 30 µm coronal sections using a cryostat (Cryostar NX50, Thermo Fisher Scientific) and sorted into 6 anterior–posterior series prior to storage.

### Histology

Immunohistochemistry: Free-floating 30 µm sections were blocked for one hour at room temperature using 2% horse serum in PBS with 0.2% Tween. Section were then incubated in anti-rat MHC-II (OX-6) (1:1000, BD Biosciences 395603) overnight at 4 °C. After three 5-min PBS washes, sections were incubated in horse anti-mouse secondary antibody (1:500, Invitrogen 31806). After three 5-min washes, DAB-mediated IHC with ABC amplification (Thermo Fisher Scientific) was used for signal detection. Stained sections were mounted onto slides and air dried prior to dehydration using a graded ethanol and xylene series after which they were cover-slipped using Depex mounting medium. Thionin: Pre-mounted 30 µm sections were stained using Thionin (1.25 g/L) to delineate lesion boundaries. Immunofluorescence: for tissue staining pre-mounted 10 µm sections were used, for cell staining 5 × 10^4^ cells were seeded onto coverslips. Tissue or cell samples were blocked for one hour at room temperature using 10% donkey serum in PBS with 1% Triton-X-100 and 1% Tween. Sections were then incubated overnight at 4 °C with TMEM119 (1:500, Proteintech CL-64766948) and CD14 (1:200, Bioss 1192R) or Iba1 (1:500, Wako 019–19741) in blocking solution. After three 5-min PBS washes CD14 or Iba1 samples were incubated in donkey anti-rabbit 488 conjugated secondary antibody. Finally, slides were washed three times with PBS prior to being cover-slipped with DAPI anti-fade aqueous mounting medium.

### Microscopy analysis

Slides were visualized using brightfield or fluorescent microscopy (Nikon Eclipse Ni-E, Nikon DS Fi2 colour camera, Nikon Qi2 fluorescent camera, NIS Elements Imaging) and example images were captured using × 10 and × 20 objectives.

### Stroke volume quantification

Stroke regions were outlined within an anterior–posterior series of sections stained with Thionin on each section within a series where the lesion was visible and quantified using Image J [44]. An example of the identified stroke regions is provided in Additional file 1: Fig. S3. The ipsilateral and contralateral hemispheric areas were measured to correct for edema and the stroke area from each section was multiplied by the section thickness to calculate volume.

### Statistical analyses

All statistical analysis was performed using Graphpad Prism 8 software. Within animal comparisons were performed using Wilcoxon paired rank test, group comparisons were performed using unpaired Student’s *t*-test or Mann–Whitney test with a significance value of *p* = 0.05.

### Study approval

All animal procedures were approved by the Animal Care Committee at Western University (protocol 2018-132). All rats used in this study were housed in facilities maintained by Western University Animal Care and Veterinary Services.

## Results

### In vitro validation of microglia EV targets

**Fig. 1:**
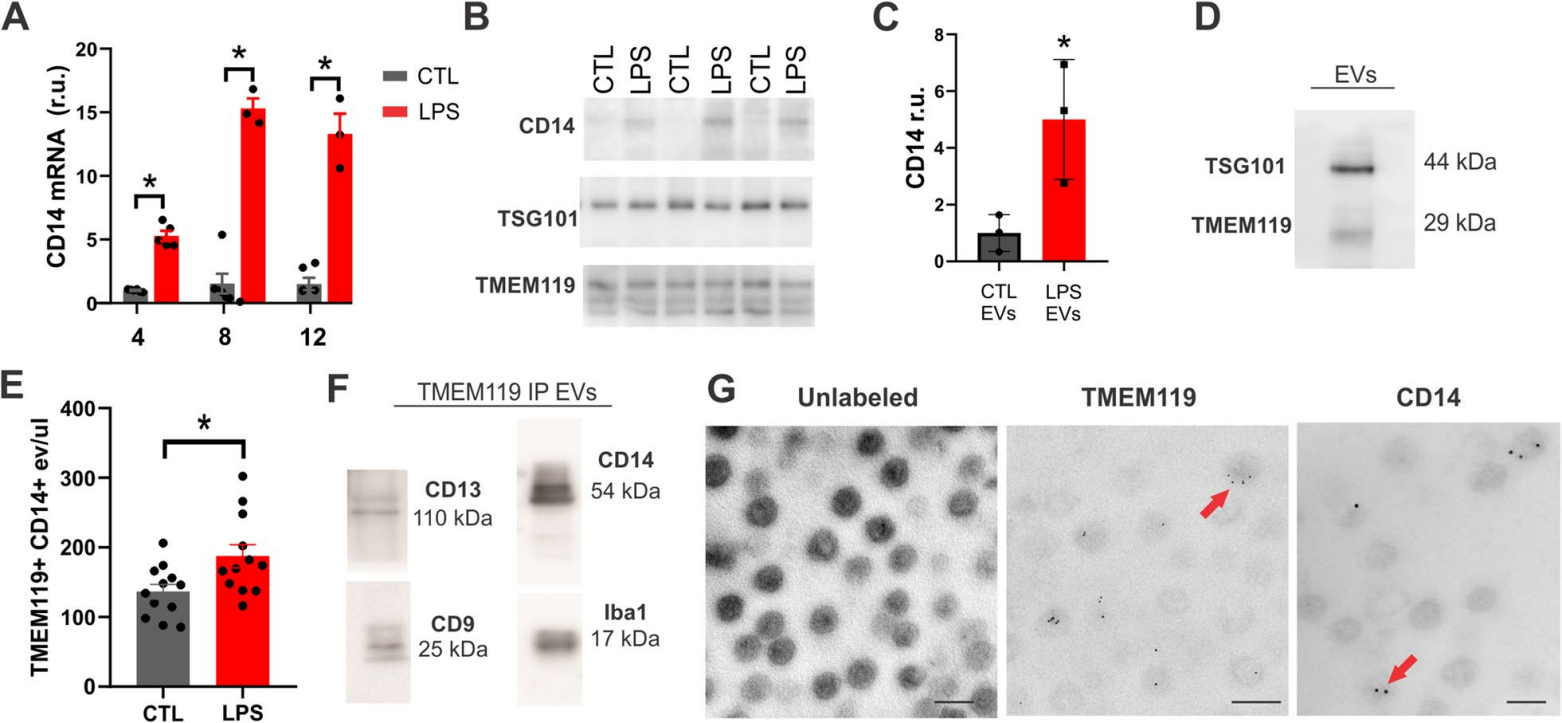
TMEM119^+^ CD14^+^ EV release is increased from activated microglia. **A** qPCR of CD14 expression following LPS treatment of BV-2 microglia. **B** Western blot of CD14, TMEM119 and TSG101 in EVs isolated from BV-2 microglia supernatant 24 h with or without LPS treatment (500 ng/mL). **C** Quantification of CD14 expression on EVs isolated from BV-2 microglia supernatant 24 h with or without LPS treatment (N = 3 separate experiments) *indicates *p* < 0.05 measured using Student’s *t*-test. **D** Western blot of TMEM119 and TSG101 on EVs isolated from BV-2 microglia supernatant. **E** Quantification of TMEM119^+^/CD14^+^ EVs in supernatant 24-h post-LPS treatment measured using nanoflow cytometry. **F** Immunoprecipitation of TMEM119^+^ EVs out of rat plasma demonstrates co-localization of CD14, CD13, CD9, microglia protein Iba1. **G** Transmission electron microscopy of unlabeled EVs and immuno-gold mediated labeling of TMEM119 and CD14 on EVs isolated from BV2-microglia 24 h post-LPS treatment. Magnification 46,000 scale bar indicates 200 nm (left), 92,000 (centre and right), scale bar indicates 100 nm

**Fig. 2:**
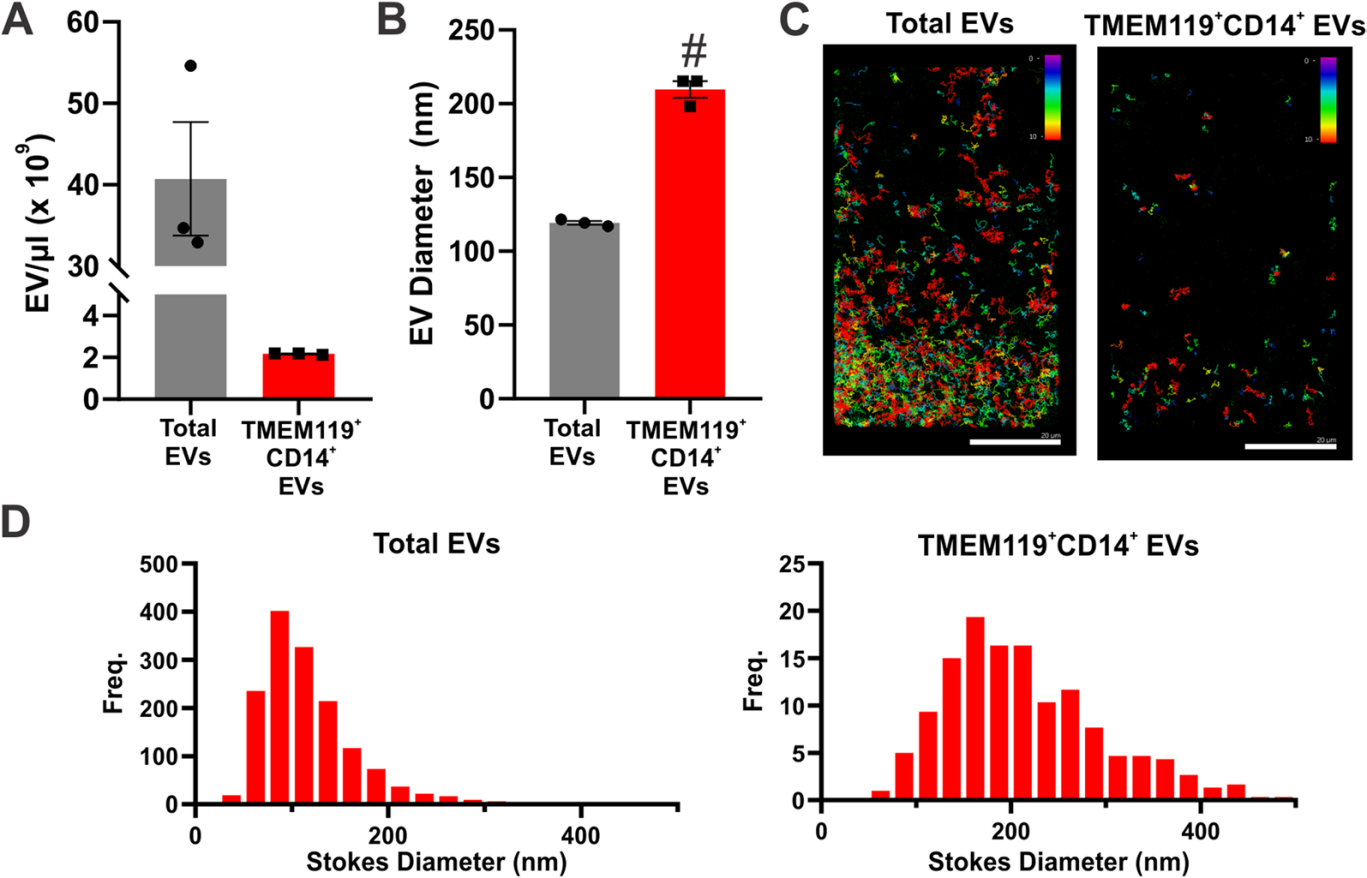
Characterization of rat plasma derived TMEM119^+^/CD14^+^ EVs **A** Concentration and **B** average size of total EV population and dual-immunoprecipitated TMEM119^+^/CD14^+^ EVs from three post-stroke plasma samples **C** Fluorescent nanoparticle tracking images of total EV population and dual-immunoprecipitated TMEM119^+^/CD14^+^ EVs stained with membrane dye (Exoglow-488). Colour scale indicates track length of measured particles **D** Size distribution of total EV population and dual-immunoprecipitated TMEM119^+^/CD14^+^ EVs

To confirm alterations to MEVs following change in cellular phenotype, BV-2 microglia were exposed to LPS to stimulate a pro-inflammatory response. In response to LPS, BV-2 microglia significantly upregulated CD14 expression (Fig. 1A). Both 100 ng/mL and 500 ng/mL LPS were sufficient to increase CD14 expression, however 500 ng/mL elicited a more robust expression change (Additional file 1: Fig. S4). This increase was reflected on EVs isolated from the media, 24 h post LPS exposure (Fig. 1B, C). The EVs isolated from cell culture supernatant also expressed the microglia protein TMEM119 (Fig. 1D) which remained constant regardless of exposure to LPS (Fig. 1B). In BV-2 microglia and primary microglia TMEM119 expression co-localized with Iba1 and remained stable after 24 h LPS exposure (Additional file 1: Fig. S5). To investigate whether the increase of CD14 expression on EVs can be detected using nanoflow cytometry, TMEM119^+^ and CD14^+^ particles in supernatant were fluorescently labelled with antibodies 24 h after LPS exposure. Results demonstrated a significant increase in TMEM119^+^/CD14^+^ EVs in culture media following LPS exposure (187.4 eV/µL ± 16.46) in comparison to control samples (136 eV/µL ± 10.63, *p* = 0.0161) (Fig. 1E). To confirm the presence of TMEM119^+^/CD14^+^ EVs in plasma we performed co-immunoprecipitation using TMEM119^+^ EVs isolated from 28-day post-stroke rat plasma. EVs immunoprecipitated using antibodies against TMEM119 demonstrated co-localization of CD14, the microglia protein Iba1, CD9, and CD13 which has previously been reported to be enriched on MEVs (Fig. 1F) [45]. To validate localization of EV markers, TEM of EVs isolated from cell culture medium demonstrated TMEM119 and CD14 presence on vesicles using immuno-gold labelling (Fig. 1G). To quantify the number of TMEM119^+^/CD14^+^ EVs in the plasma, EVs were isolated using double immunoprecipitation followed by fluorescent NTA to determine size and concentration (Fig. 2). TMEM119^+^/CD14^+^ EVs have a concentration of 2.16 × 10^9^ ± 2.98 × 10^7^ (Fig. 2A) and an average diameter of 209.70 nm ± 9.86 nm which was significantly larger than the average diameter of the total EV population (119.3 nm ± 2.24 nm) (*p* = 0.0001) (Fig. 2B). The size distribution of the total EV and TMEM119^+^/CD14^+^ EV populations is depicted in Fig. 2D.

### TMEM119^+^/CD14^+^ EVs increase specifically post-stroke in the rat

**Fig. 3:**
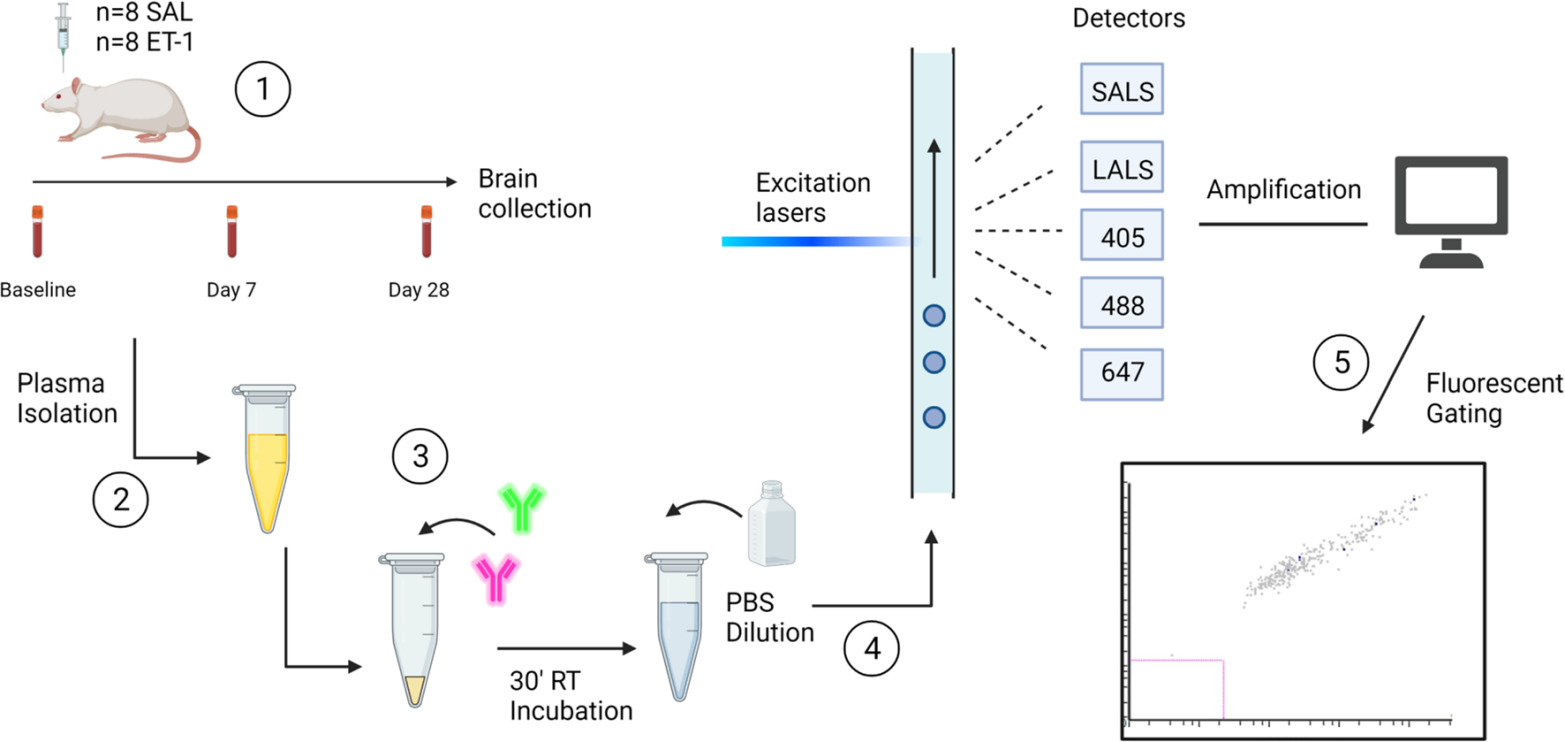
Experimental overview. (1) Experimental model of ischemia with blood collections prior to, or 7 and 28 days following injection of saline or Endothelin-1 into the dorsal striatum (2) Isolation of plasma and storage at − 80 °C until further analysis (3) 10 μL of plasma is incubated with target antibody for 30 min prior to dilution with PBS and nanoflow cytometry analysis (4) EVs pass through lasers with wavelengths of 405 nm, 488 nm and 638 nm and detectors measure SALS, LALS and fluorescent light scatter (5) Size and fluorescent gating are used to generate outputs of the target EV population. Figure created with Biorender.com

**Fig. 4:**
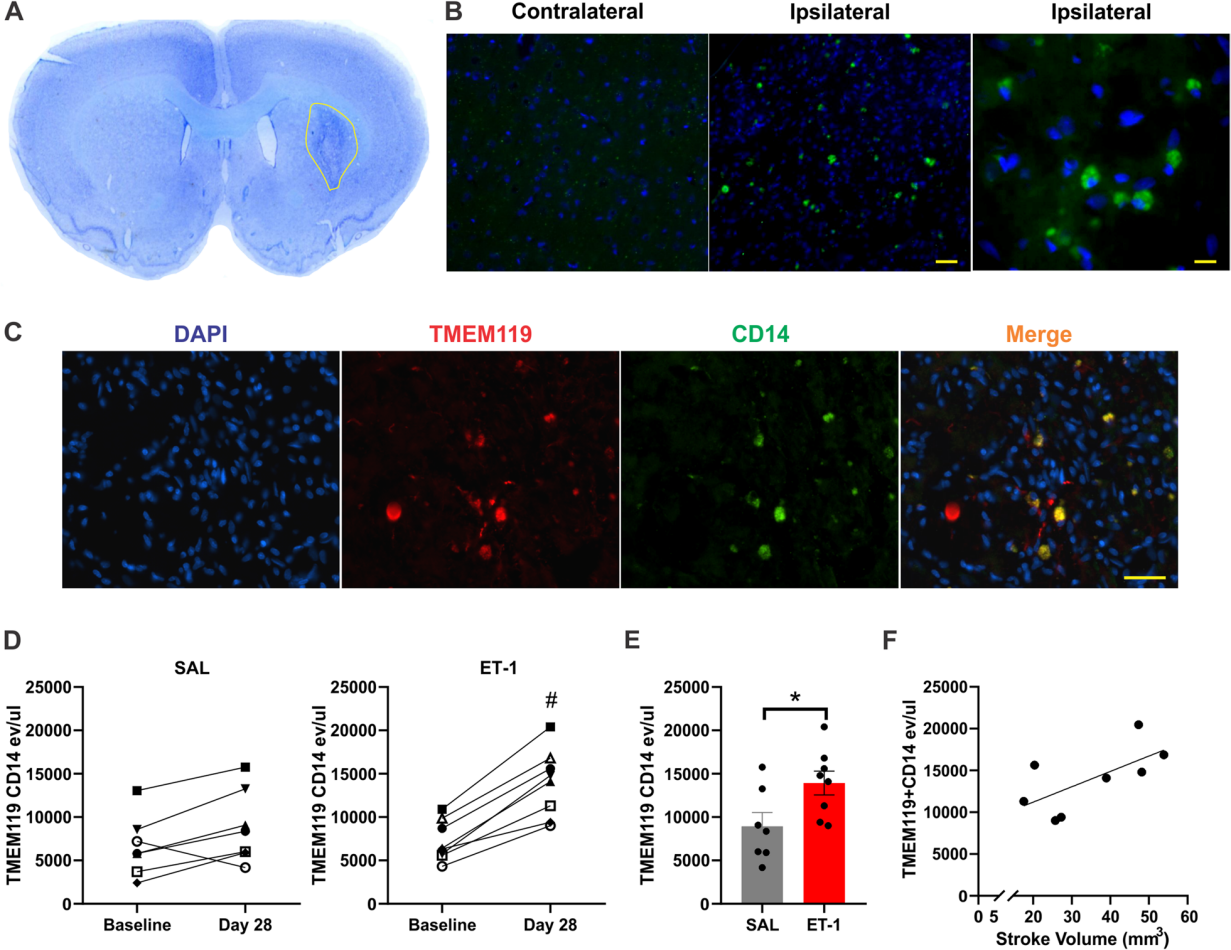
TMEM119^+^ CD14^+^ EVs are significant increased 28 days following ET-1 injection. **A** Example of thionin staining of stroke region **B** IF of CD14-488 in contralateral and ipsilateral striatum of ET-1 injected animal. Images taken at × 10 and × 40 magnification, scale bar indicates 100 µm (centre) and 50 µm (right). **C** IF of TMEM119 (647) and CD14 (488) within stroke region of ET-1 injected animal. Images taken at 20 × magnification, scale bar indicates 100 µm **D** TMEM119^+^/CD14^+^ labelled events/uL in plasma from baseline and 28-day samples of saline and ET-1 injection groups. **E** TMEM119^+^/CD14^+^ labelled events/μl of plasma from 28-day samples of ET-1 and saline injection groups **F** Correlation between TMEM119^+^/CD14^+^ EVs and stroke volume. # Indicates statistical significance (*p* < .05) measured using Wilcoxon paired rank test. *Indicates statistical significance (*p* < .05) measured using Mann–Whitney test

To measure alterations of microglia-derived EVs, plasma from saline and ET-1 injected rats was analyzed using nanoflow cytometry 7- and 28-days post-surgery. An overview of the study design and methodological approach is provided in Fig. 3. Figure 4A depicts a stroke region from an ET-1 injected animal. Immunofluorescence histochemistry confirmed co-expression of CD14 and TMEM119 within the stroke-induced striatum, with no detectable expression of CD14 within the contralateral striatum (Fig. 4B, C). In the ET-1 injected rats, nanoflow cytometry of dual-labelled TMEM119^+^/CD14^+^ events/μL of plasma were significantly increased systemically 28 days post stroke (13,925 eV/ µL ± 1372) in comparison to baseline samples collected from the same rats (7238 eV/µL ± 816.1, *p* = 0.0078) and 28-day samples from the saline injected control rats (8933 eV/µL ± 1591, *p* = 0.0401) (Fig. 4D, E). There was no significant difference in TMEM119^+^/CD14^+^ EVs in the saline control group between baseline and 28-day plasma samples (Fig. 4D). TMEM119^+^/CD14^+^ events in 28-day samples of ET-1 injected rats were weakly correlated with infarct volume (*r* = 0.66, *p* = 0.0775) which ranged from 17.5 to 53.8 mm^3^ (Fig. 4F). To estimate size of TMEM119^+^/CD14^+^ EVs measured using nanoflow cytometry, they were compared to standardized silicon and polystyrene beads of known diameter. TMEM119^+^/CD14^+^ EVs in plasma produce light scatter profiles that overlap with beads 110–300 nm in diameter (Additional file 1: Fig. S1). TMEM119^+^/CD14^+^ events were not significantly different between baseline and 7-day samples in either surgical group (Additional file 1: Fig. S6).

### TMEM119^+^ MHC-II^+^ EVs increase 28-days following ET-1 and saline injection

**Fig. 5:**
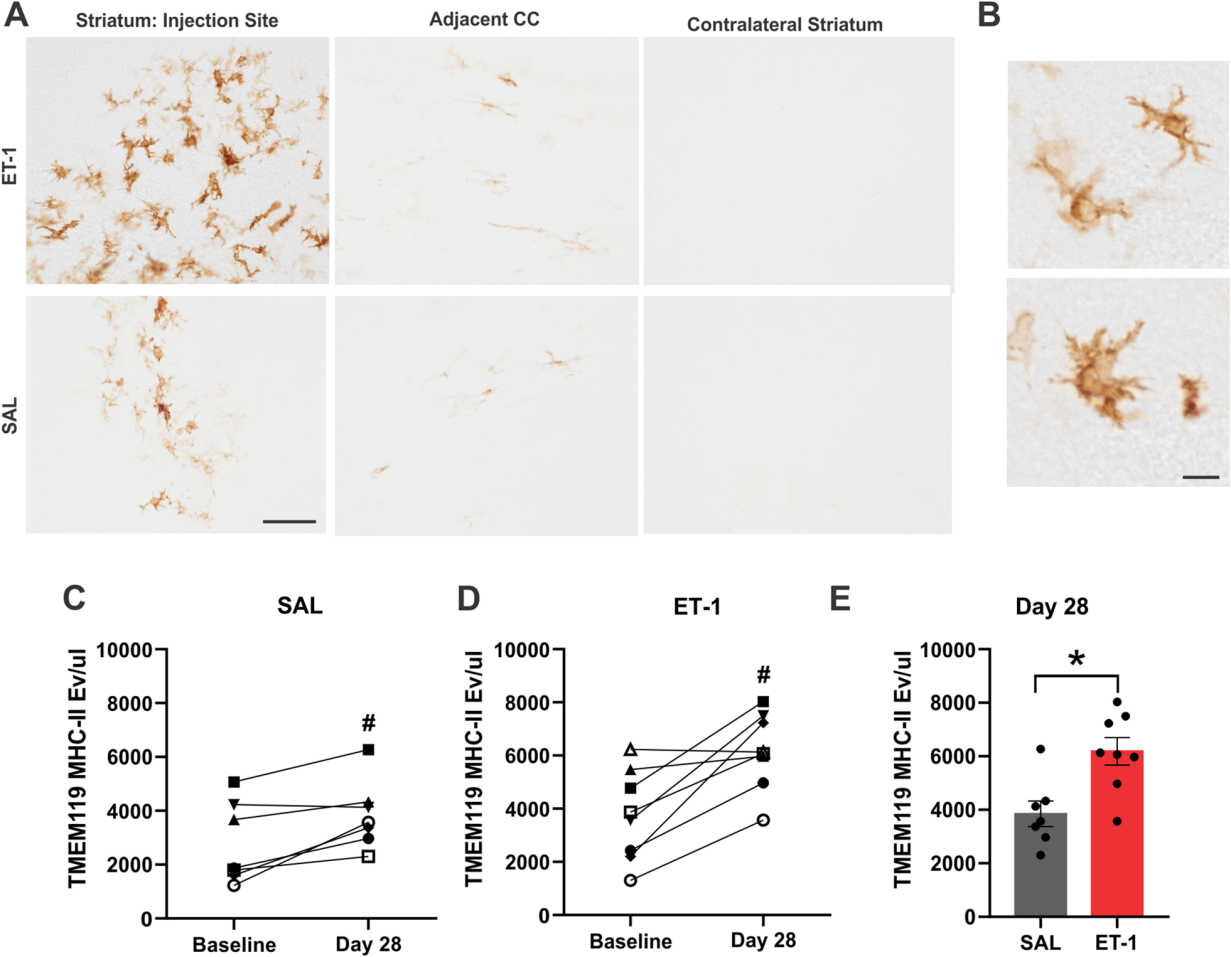
TMEM119^+^/MHC-II^+^ EVs are significant increased 28 days following ET-1 injection. **A** MHC-II IHC in selected regions of ET-1 and saline injected animals: injection site, adjacent corpus callosum (CC) and contralateral striatum. Scale bar indicates 100 µm. **B** Images taken at × 40 magnification depicting microglia morphology within stroke region, scale bar indicates 25 μm **C** TMEM119^+^MHC-II^+^ labelled events/μL of plasma from baseline and 28-day samples of saline injection group. **D** TMEM119^+^MHC-II^+^ labelled events/μL of plasma from baseline and 28-day samples of ET-1 injection group. **E** TMEM119^+^/MHC-II^+^ labelled events/µL of plasma from 28-day samples of ET-1 and saline injection groups. # Indicates statistical significance (*p* < 0.05) measured using Wilcoxon paired rank test. *Indicates statistical significance (*p* < 0.05) measured using Mann–Whitney test

In addition to CD14, MHC-II expression is significantly increased on microglia in the response to stroke and it’s expression on the surface of EVs has been well characterized [19, 46]. Therefore, circulating levels of TMEM119^+^/MHC-II^+^ EVs were also evaluated at baseline, 7- and 28-days post-stroke. Immunohistochemistry of MHC-II^+^ cells confirmed dense clusters of microglia within the stroke-induced striatum with sparse MHC-II microglia in the neighboring corpus callosum (CC), as compared to minimal detection of MHC-II^+^ microglia within the saline injected control (Fig. 5A). TMEM119^+^/MHC-II^+^ EVs measured 28-days following saline injection were elevated (3849 eV/µL ± 479.2) in comparison to baseline samples (2781 eV/µL ± 571.6, *p* = 0.0313) (Fig. 5B). TMEM119^+^/MHC-II^+^ EVs were significantly increased in plasma samples 28 days post-stroke (6184 eV/µL ± 510.6) in comparison to baseline samples (3730 eV/µL ± 602 *p* = 0.0156) and saline injected rats (Fig. 5C, D). There was no significant difference between TMEM119^+^/MHC-II^+^ EVs measured at baseline versus day 7 in either surgical group (Additional file 1: Fig. S6).

## Discussion

Persistent inflammation driven by pro-inflammatory microglia in the chronic stage post-stroke is associated with exacerbated tissue damage and worse neurological outcomes [14–18]. However, methods to detect ongoing microglial activation post-stroke are invasive and non-specific. To address this technical gap, we identified novel circulating EV biomarkers of microglia activation using a preclinical stroke model with an acute and measurable increase in microglial activation. This represents the first study to our knowledge to report plasma-based detection of MEVs specific to activation status. Furthermore, the development of a nanoflow cytometry approach enables direct measurement of circulating EVs without the need for time-intensive and variable EV isolation steps.

Since circulating EVs originate from any cell-type in the body, a combination of markers is required to improve confidence in cellular origin and in the case of microglia, activation status. This is further complicated by the similarity between microglia and peripheral macrophages due to their shared monocytic lineage [47]. Using the BV-2 microglial cell line, we validated the enrichment of the surface marker TMEM119 on MEVs in comparison to peripheral macrophages. This is consistent with previous studies reporting TMEM119^+^ EVs isolated from total brain homogenates and CSF [36]. Due to the difficulty of obtaining sufficient yield from adult microglia, and the lack of TMEM119 expression in neonatal primary microglia [48], EV experiments from microglia cell culture supernatants were limited to BV-2 microglia. However, TMEM119 expression was confirmed in primary microglia isolated from adult rats suggesting that like BV-2 microglia their EVs would also contain TMEM119 (Additional file 1: Fig. S2). Although TMEM119 is considered a microglia-specific marker in comparison to other brain cells, it is also expressed by dendritic cells and osteoblasts which supports the need for co-markers to improve specificity of a potential plasma-based assay of TMEM119^+^ MEVs.

CD14 is a co-receptor for Toll-like receptor 4 (TLR4) [49], both of which increase following microglia activation and play a role in the microglia response to ischemia after they are activated by endogenous damage associated molecular patterns (DAMPs) such as ATP released from dying neuron [50–53]. In experimental stroke models, CD14 expression is increased 7 and 28 days post-stroke, and has been documented in post-stroke human microglia within the lesion site [54–56]. Furthermore, recent proteomic studies have demonstrated that CD14 expression is increased on MEVs following pro-inflammatory stimuli such as ATP [37]. Therefore, CD14 represents a strong activation-specific candidate for an MEV co-marker, especially since it is expressed at low levels in dendritic cells in comparison to macrophages and is not expressed by osteoblasts [57]. Our in vitro experiments confirmed upregulated expression of CD14 on EVs isolated from cell culture supernatant following LPS exposure (Fig. 1). Electron microscopy confirmed the presence of both TMEM119 and CD14 on the surface of EVs, which supports their use as candidate targets for nanoflow cytometry labeling. Finally, we validated that CD14 and TMEM119 are dually expressed on a population of EVs in the plasma using a combination of immunoprecipitation, western blot, and nanoparticle tracking analysis. Using nanoflow cytometry, TMEM119^+^/CD14^+^ EVs were directly labelled in the plasma and demonstrate an increase 28-days post-stroke, but not 7-days post-stroke. This increase may be due to both the upregulation of CD14 expression by microglia as well as local proliferation of microglia that occurs post-stroke, both of which would results in an increase in CD14^+^ MEV release. TMEM119^+^/CD14^+^ EVs were weakly correlated with stroke volume suggesting that the extent of tissue damage may be reflected by circulating microglial-EVs, however this finding requires further validation with a larger sample size.

MHC-Class II represents another marker that is expressed on EVs released from macrophages and other immune cells after pro-inflammatory signaling [46, 58]. Post-stroke there is an abundance of MHC-II expressing microglia in the infarct region and neighboring white matter tracts which persists into the chronic stage [14, 27]. Importantly, in a recent study this phenotype of microglia was not reliably detected using the TSPO-PET tracer used frequently as a measure of neuroinflammation [27]. Therefore, we investigated whether measuring MHC-II on plasma MEVs could serve as a marker of post-stroke microglia activation. Although other cells in the infarct region, such as infiltrating macrophages or perivascular macrophages may express MHC-II, these populations do not express significant levels of TMEM119 [48, 59, 60]. TMEM119^+^/ MHC-II^+^ EVs were significantly increased at 28-days in the control saline injections (likely due to mechanical injury) and even more so in the ET-1 injected stroke rats. Histology confirmed the presence of MHC-II^+^ activated microglia within in the injection site of both the saline animals and ET-1 animals, demonstrating the sensitivity of nanoflow cytometry to changes in MHC-II^+^ MEV release.

In the plasma assays of both TMEM119^+^/CD14^+^ and TMEM119^+^/ MHC-II^+^ EVs, dual-labelled EVs were increased at 28 days post-stroke, but insignificantly at 7-days post-stroke. For this we have two potential explanations. Expression levels of TMEM119 are downregulated in experimental models of acute inflammation such as experimental autoimmune encephalomyelitis (EAE). It is possible that immediately post-stroke, the expression levels of TMEM119 are lower in microglia, which have then recovered by day 28. The temporal expression pattern of TMEM119 post-stroke has not been well characterized and further work is necessary to determine its reliability in the acute phase of neurological injury. The second hypothesis relates to the rate of MEV release based on cellular activity. Although the number of pro-inflammatory microglia in the lesion site increases until 7–14 days post-stroke, we do not know that MEV release follows the same trajectory [13, 61]. It has been suggested the microglia release EVs as a means of resolving their pro-inflammatory state, as impairing this process prevents resolution of microglia activation [62]. The increased signal observed at 28-days could be due to the resolving of pro-inflammatory activity by microglia. Conversely, it is known that pro-inflammatory stimulus increases macrophage EV (and presumably MEV) release in the acute phase, therefore including a 24- or 48-h timepoint in future studies is warranted to measure MEVs during this initial response.

### Limitations and future directions

Although TMEM119 distinguishes microglia and peripheral macrophages under homeostatic conditions, following ischemia infiltrating peripheral macrophages infiltrating take on a “microglia-like” state and become virtually indistinguishable from resident microglia proliferating locally. However, their infiltration has been reported to peak 4–5 days post-injury and decline afterwards suggesting the signal by day 28 is likely reflective of resident microglia which predominate [10, 63]. Furthermore, if macrophages do persist in a microglial phenotype, distinguishing between the two is less crucial as they are both contributing to the chronic post-stroke pro-inflammatory response. What remains important to distinguish is whether unique EVs populations originate from microglia of different phenotypes. While TMEM119^+^/CD14^+^ and TMEM119^+^/MHC-II^+^ EVs may reflect a phenotype upregulated during pro-inflammatory signaling, it is likely that a variety of marker combinations could be used to differentiate microglia activity. Following stroke, spatiotemporal differences in microglia phenotype can be identified by their gene expression profiles and cellular morphology [64–66]. Further elucidating the relationship between circulating EVs and specific microglia phenotypes that vary based on timing, sex and age represents an important future direction.

In this study, the type of EV subpopulation that is TMEM119^+^/CD14^+^ can’t be confirmed as the techniques employed for EV measurement or isolation (nanoflow cytometry and ultracentrifugation) do not discriminate between exosomes or other microvesicles. Nanoflow cytometry of silicon and polystyrene calibration beads indicate that TMEM119^+^/CD14^+^ EVs primarily scatter in the range of 110–300 nm beads. Although the different refractive indices of silicon/polystyrene beads in comparison to biological membranes hinders size interpretation of nanoflow cytometry results, immunoprecipitation followed by NTA confirmed that TMEM119^+^/CD14^+^ EVs fell in the 200–300 nm range. These results suggest that the TMEM119^+^/CD14^+^ EV population may be primarily composed of microvesicles derived from the cell surface membrane which are known to be larger in size than exosomes [67], although further studies need to be done to fully confirm this.

The point at which microglial activation post-stroke becomes harmful versus beneficial is not well understood. In vitro models demonstrate a protective effect of microglia on neurons in the immediate hours following hypoxia [68]. Conversely, in vitro models show that TLR4 mediated activation of microglia can facilitate neuronal death, suggesting that day-28 persistence of TLR4-CD14 activation in microglia may be an ominous sign [69]. Experimental studies have demonstrated positive effects of inhibiting microglia activation on infarct volumes and cognitive outcomes [70], although these findings have not translated as well into human populations. The success of any future anti-microglial therapy for acute neurological injury such as stroke may rely on the ability to identify individuals with persistent microglial activation. A plasma based MEV marker provides a rapid, and non-invasive platform for the measurement of microglial activity in vivo, presenting the opportunity to identify individuals with ongoing microglia activation, optimize potential therapeutic windows post-stroke, and measure effectiveness of future microglia-modulating therapeutics. This approach is not limited to the post-stroke setting, as microglia display heterogenous phenotypes in aging and neurological diseases which are being increasingly recognized due to advancements in single-cell transcriptomic approaches. This suggests that multiple MEV profiles are possible, each bearing surface markers and carrying cargo related to the provoking stimulus. While this study focused on the post-stroke alterations to MEVs, and the involvement of CD14 and MHC-II in the response to ischemic stress, future studies should harness recent advancements in transcriptomics to identify disease-specific candidate MEV markers.

## Supplementary Information

**Additional file 1:** **Table S1.** Genbank accession number and primer sequences used for qPCR experiments. **Figure S1.** Nanoflow cytometry detection of TMEM119+/CD14+ EVs in comparison to standardized bead sizes. **Figure S2.** Transmission electron microscopy images of unlabeled EVs and CD14 or TMEM119 labelled EVs (via gold-conjugated secondary antibodies). **Figure S3.** A) Anterior to posterior boundaries of lesion following ET-1 injection delineated using thionin staining. B) Lesion areas demarcated within yellow ROI. **Figure S4.** qPCR of CD14 expression following LPS treatment of BV-2 microglia at either 100 ng/ml or 500 ng/ml for either 8 or 12 hours. **Figure S5.** Immunofluorescent staining of Iba1 and TMEM119 in BV-2 microglia and primary adult microglia with and without LPS exposure (24 hours, 500 ng/ml) Scale bar indicates 100 µm. **Figure S6.** Nanoflow cytometry measurement of TMEM119+/CD14+ and TMEM119+/MHC-II+ EVs in plasma samples from saline or endothelin-1 injected rats at baseline and 7 days post-surgery. **Figure S7.** Western Blot imaging files.

## Data Availability

The datasets used and/or analysed during the current study are available from the corresponding author on reasonable request.

## Abbreviations

ATP: Adenosine tri-phosphate
CD: Cluster of differentiation
CNS: Central nervous system
DAMP: Damage-associated molecular pattern
ELISA: Enzyme-linked immunosorbent assay
ET-1: Endothelin-1
EV: Extracellular vesicles
IP: Immunoprecipitation
LALS: Long angle light scatter
LPS: Lipopolysaccharide
MEV: Microglial extracellular vesicles
MHC-II: Major histocompatibility complex
SALS: Short angle light scatter
TEM: Transmission electron microscopy;
TLR4: Toll-like receptor 4
TMEM119: Transmembrane protein 119
