# Supplementary material for: Plasma derived extracellular vesicle biomarkers of microglia activation in an experimental stroke model
Source: J Neuroinflammation. 2023 Jan 31;20:20. doi: 10.1186/s12974-023-02708-x (PMC9890769; doi:10.1186/s12974-023-02708-x)

**Table S1:** Genbank accession number and primer sequences used for qPCR experiments.

| Gene | Forward Primer (5”🡪3”) | Reverse Primer (5”🡪3”) |
| --- | --- | --- |
| CD14 (mus musculus) | AATTTACAGGGGCTGCCGAA | TGAAAGCGCTGGACCAATCT |
| RPl13α (mus musculus) | GTTCGGCTGAAGCCTACCAG | TTCCGTAACCTCAAGATCTGCT |

**Figure S1: Nanoflow cytometry detection of TMEM119^+^/CD14^+^ EVs in comparison to standardized bead sizes**. A-B) Scatter plot of silicon beads (180 nm, 240 nm, 300 nm &590 nm) and 488-conjugated polystyrene beads (110 nm & 500 nm) C) Scatterplot of total plasma EVs D) TMEM119-647^+^ gated EVs, E) CD14-488^+^ gated EVs F) Dual positive TMEM119-647^+^ & CD14-488^+^ EVs G) PBS alone: 647-SALS, 488-SALS and dual 647-488 gated channels H) Plasma alone: 647-SALS, 488-SALS and dual 647-488 gated channels. Red boxes indicate ROIs for fluorophore gating I) TMEM119-647 antibody incubation alone with plasma: 647 gated, 488 gated and 647-488 gated channels J) CD14-488 antibody incubation alone with plasma: 647-gated, 488-gated and 647-488 gated channels

**
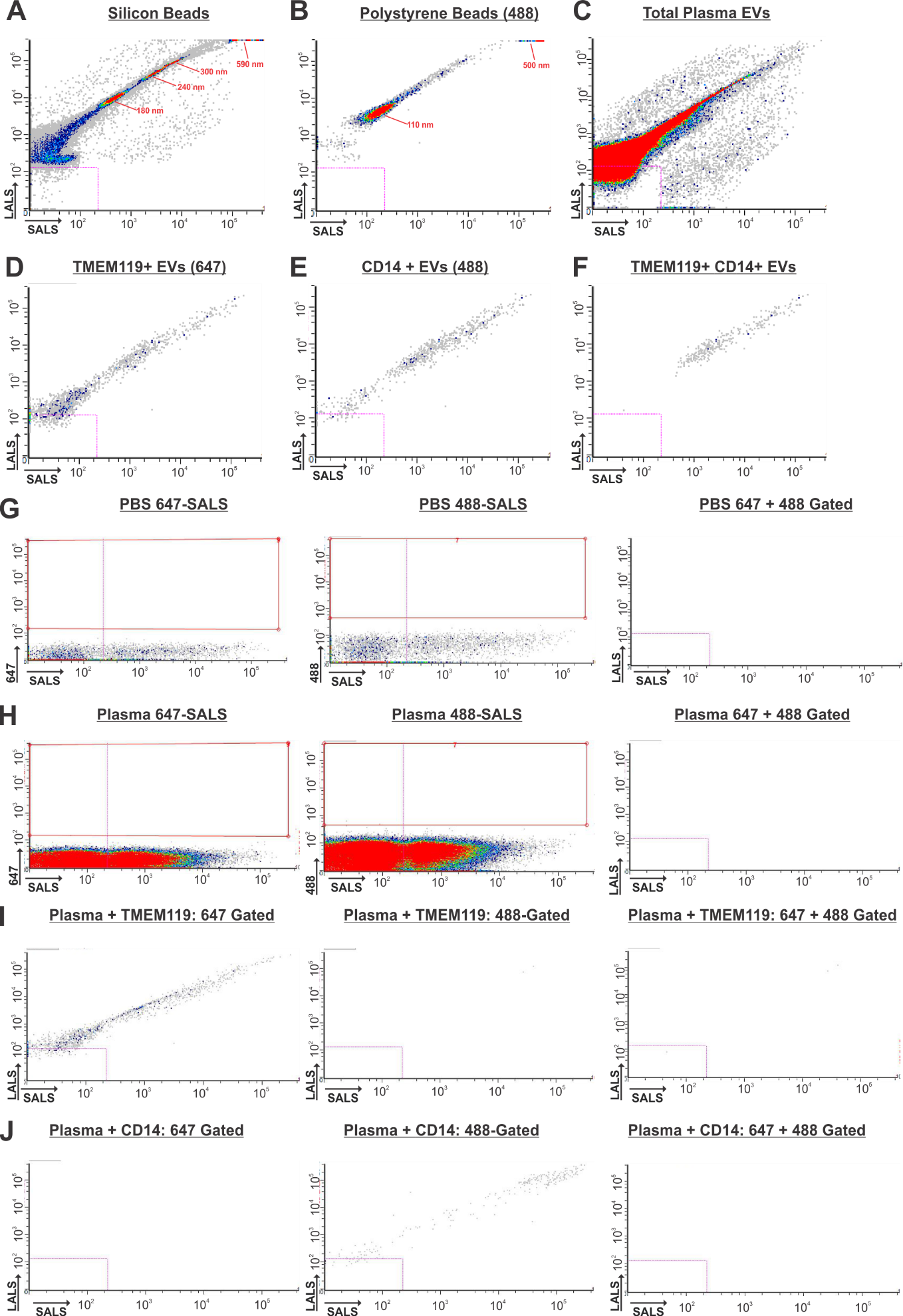
**

**Figure S2:** Transmission electron microscopy images of unlabeled EVs and CD14 or TMEM119 labelled EVs (via gold-conjugated secondary antibodies)


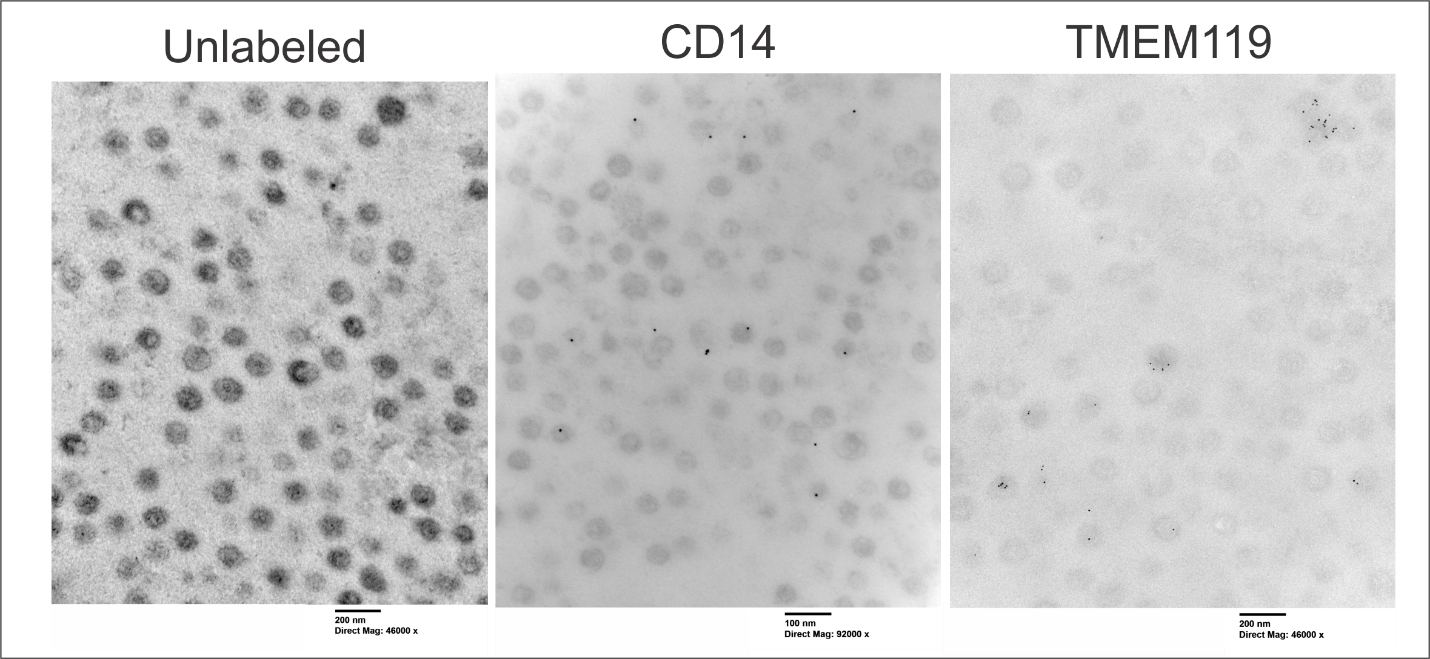


**Figure S3:** A) Anterior to posterior boundaries of lesion following ET-1 injection delineated using thionin staining. B) Lesion areas demarcated within yellow ROI.


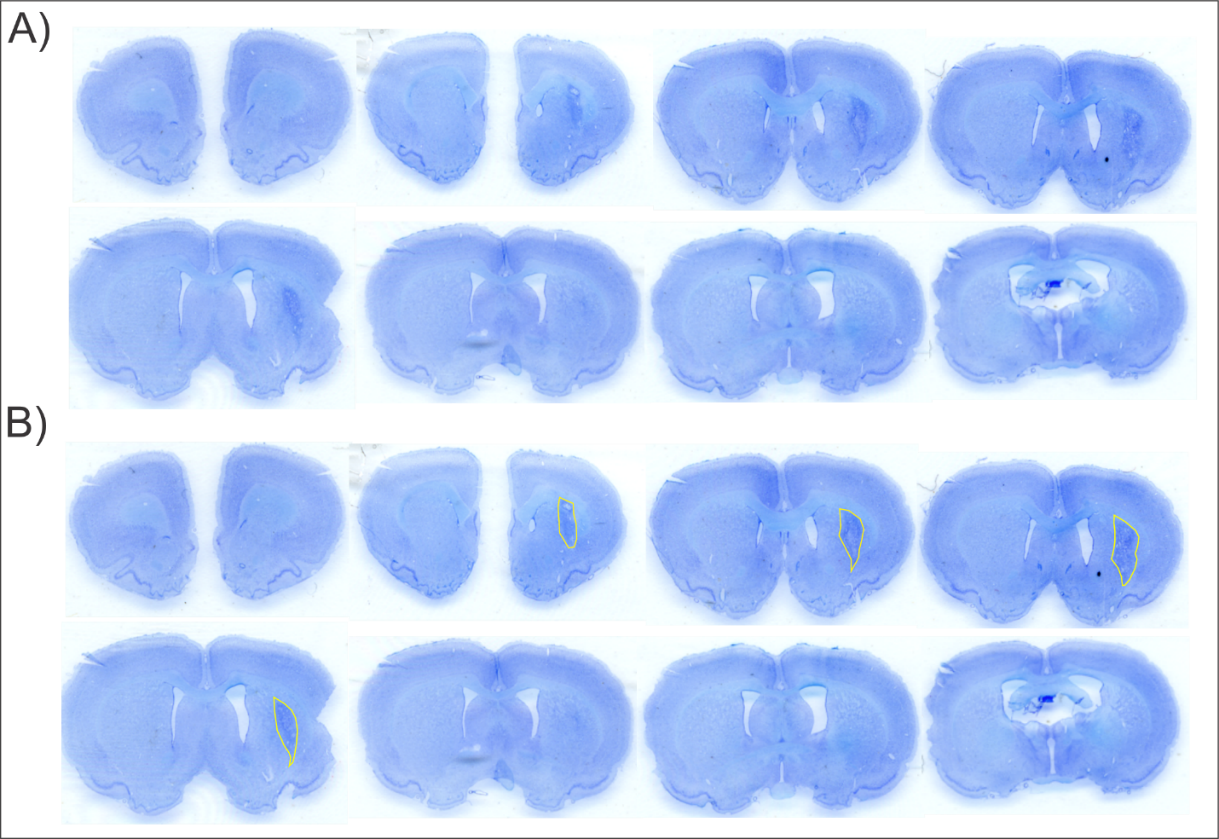


**Figure S4:** qPCR of CD14 expression following LPS treatment of BV-2 microglia at either 100 ng/ml or 500 ng/ml for either 8 or 12 hours.

**
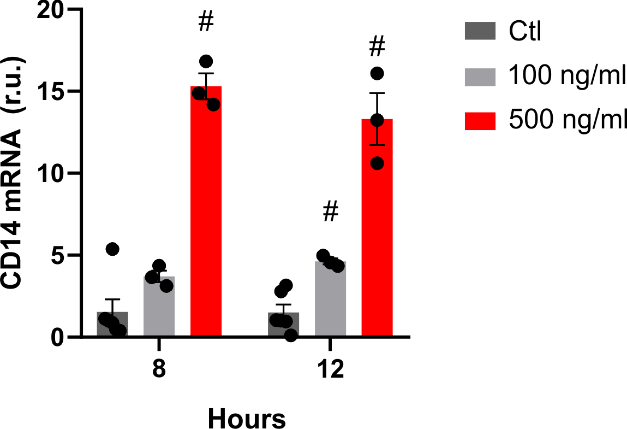
**

**Figure S5: Immunofluorescent staining of Iba1 and TMEM119 in BV-2 microglia and primary adult microglia with and without LPS exposure (24 hours, 500 ng/ml)** Scale bar indicates 100 µm.

**
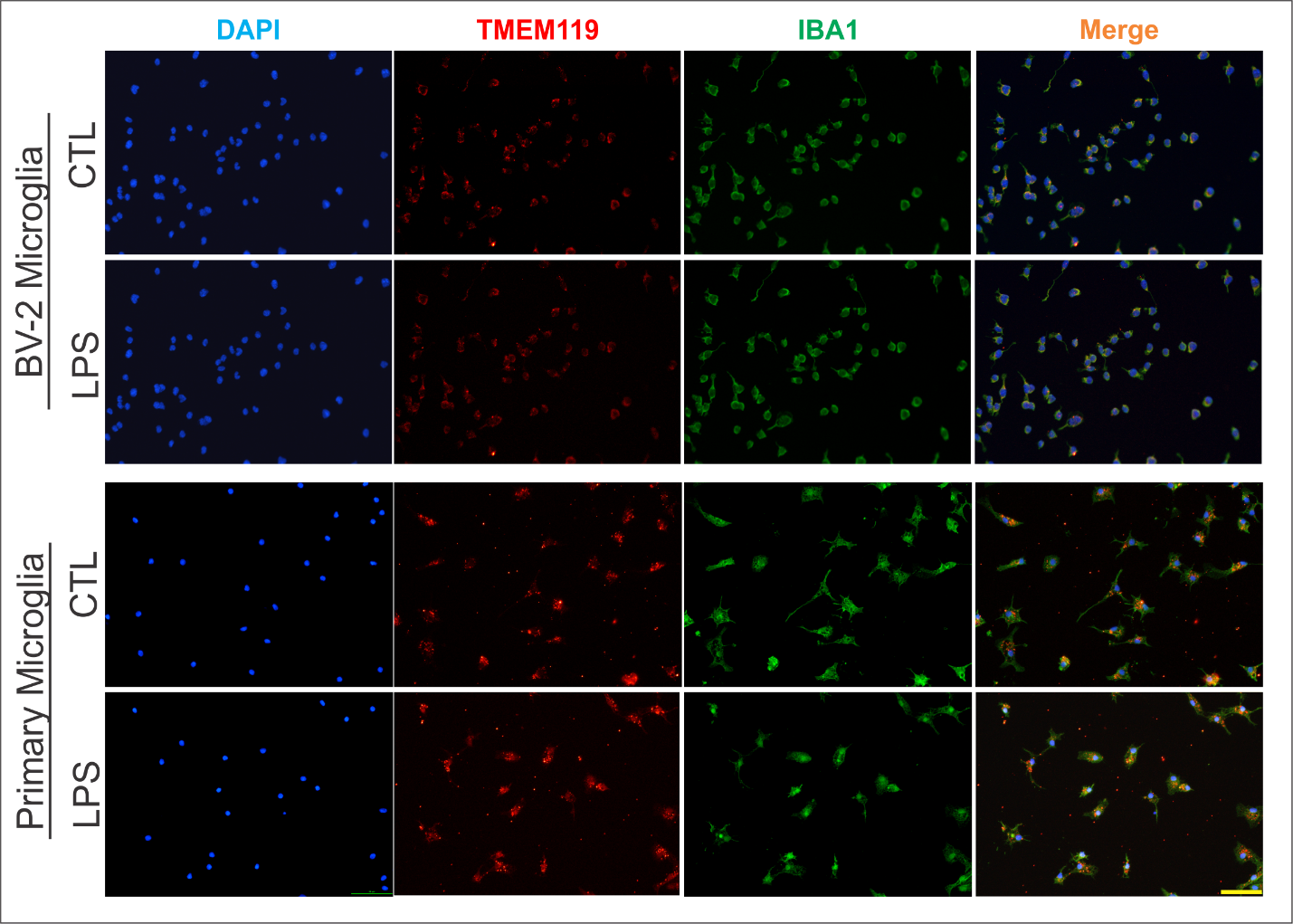
**

**Figure S6:** Nanoflow cytometry measurement of TMEM119^+^/CD14^+^ and TMEM119^+^/MHC-II^+^ EVs in plasma samples from saline or endothelin-1 injected rats at baseline and 7 days post-surgery.

**
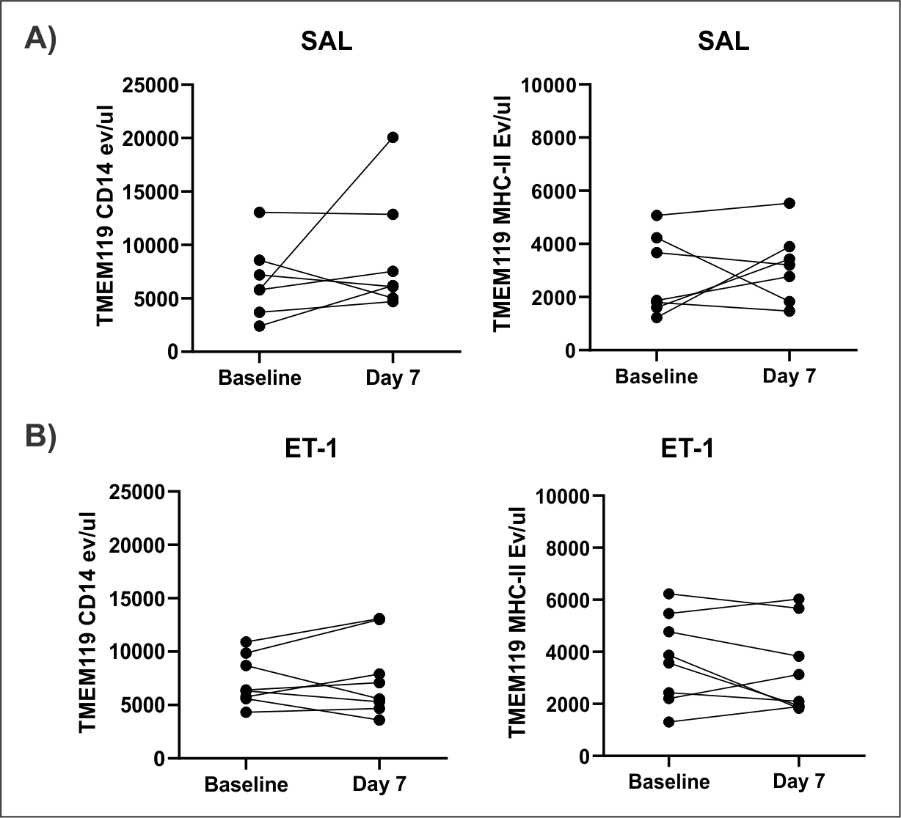
**

**Figure S7: Western Blot imaging files**


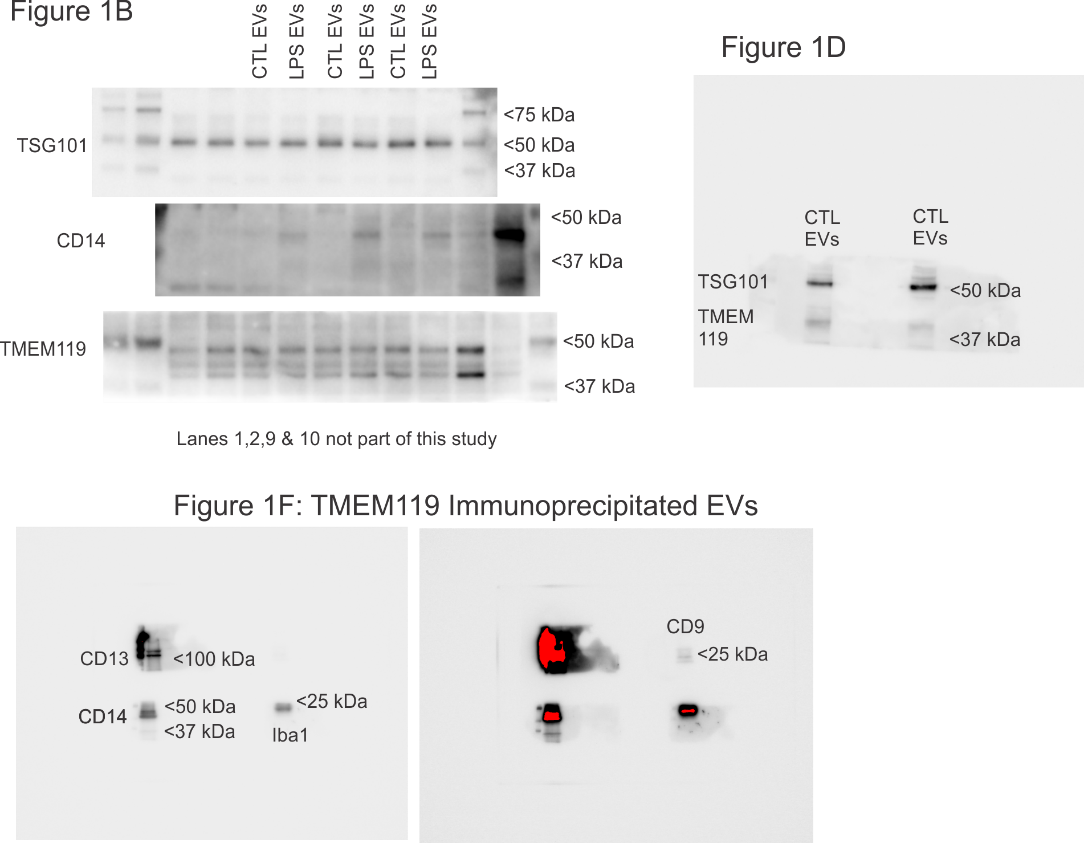

Supplement: Supplementary file 1 — Additional file 1: Table S1. Genbank accession number and primer sequences used for qPCR experiments. Figure S1. Nanoflow cytometry detection of TMEM119+/CD14+ EVs in comparison to standardized bead sizes. Figure S2. Transmission electron microscopy images of unlabeled EVs and CD14 or TMEM119 labelled EVs (via gold-conjugated secondary antibodies). Figure S3. A) Anterior to posterior boundaries of lesion following ET-1 injection delineated using thionin staining. B) Lesion areas demarcated within yellow ROI. Figure S4. qPCR of CD14 expression following LPS treatment of BV-2 microglia at either 100 ng/ml or 500 ng/ml for either 8 or 12 hours. Figure S5. Immunofluorescent staining of Iba1 and TMEM119 in BV-2 microglia and primary adult microglia with and without LPS exposure (24 hours, 500 ng/ml) Scale bar indicates 100 µm. Figure S6. Nanoflow cytometry measurement of TMEM119+/CD14+ and TMEM119+/MHC-II+ EVs in plasma samples from saline or endothelin-1 injected rats at baseline and 7 days post-surgery. Figure S7. Western Blot imaging files. [file 12974_2023_2708_MOESM1_ESM.docx]
